# Regarding “Burden of Comorbidities and Healthcare Resource Utilization Among Medicaid-Enrolled Extremely Premature Infants”

**DOI:** 10.36469/001c.73989

**Published:** 2023-04-04

**Authors:** Ava Tsapatsaris, Miran Bhima, Tejas C. Sekhar

**Affiliations:** 1 Gallatin School of Individualized Study New York University, New York, NY; 2 Trinity College of Arts and Sciences Duke University, Durham, North Carolina; 3 Division of Pulmonary and Critical Care Medicine Washington University School of Medicine, St. Louis, Missouri

**Keywords:** Medicaid, healthcare utilization, preterm birth, socioeconomic disparities, patient navigators, care management, neonatal intensive care unit, NICU, managed care organizations, MCOs

## Abstract

In their article, Mowitz et al investigated the burden of comorbidities and healthcare resource utilization among extremely premature infants enrolled in Medicaid, laying a foundation for further policy action.

Preterm birth is the leading cause of infant mortality worldwide.^1^ The most common complications include respiratory distress syndrome and bronchopulmonary dysplasia. While complications from preterm birth occur regardless of insurance status, pregnant patients insured by Medicaid are at a higher risk for unfavorable birth outcomes than pregnant patients with private insurance.^2^ In their article, Mowitz and colleagues investigated the burden of comorbidities and healthcare resource utilization among extremely premature (EP) infants enrolled in Medicaid. The authors analyzed data from publicly available Medicaid databases of 6 US states (Iowa, Kansas, New Jersey, Mississippi, Missouri, and Wisconsin) from 1997 to 2018, which included information on 26.6 million Medicaid beneficiaries. The incidence of EP births is correlated with low socioeconomic status (SES) and Medicaid enrollment. EP infants (born at 23-28 weeks of gestational age) possessed a relatively high burden of comorbidities, with more than 70% having at least 1 comorbidity. Additionally, EP infants were observed to require considerably higher use of medical resources, which involved more frequent admissions to hospitals, intensive care units, and surgeries. Disparities in care for vulnerable patient populations such as EP infants and their families underscore the need for focused interventions to engender favorable outcomes.

The higher proportion of EP births among Medicaid-insured patients in the 6 examined states may be linked to their lower SES, given that 5 of these 6 states have a median household income ranking in the bottom half of all US states. Despite quality improvement networks (eg, state partnerships) that have created more detailed recommendations for respiratory support in the EP population, Mowitz et al denote that premature infants with comorbidities who require extensive use of healthcare resources typically induce high-cost burdens upon their respective families. We suggest that the standardization of care mentioned by the authors include socioeconomically tailored approaches to meet the needs of a diverse patient population. These interventions can include (but are not limited to) patient navigators and social work methodologies.

Patients not enrolled in Medicaid are more likely to have higher educational attainment, elevated SES, and greater access to quality prenatal care compared with Medicaid patients.^2^ Since pregnant Medicaid patients receive less prenatal care than non-Medicaid pregnant patients, patient navigators play a critical role in advocacy and care coordination.^3^ Patient navigators—such as community health workers and patient care coordinators—are qualified medical or non-medical professionals who guide patients through nuanced and oftentimes fragmented medical systems.^4^ In a study of patients with heart failure, a free patient navigator program reduced rates of readmission by 8%.^5^ Patient navigators who assisted with negotiating out-of-pocket costs helped 11 186 patients with cancer save an average total of $3.5 million per year during the course of the 11-year study, or approximately $313 per patient.^6^ Implementing patient navigators to support and guide vulnerable patient populations may be instrumental in alleviating socioeconomic disparities in healthcare resource utilization.

Serving the EP infant population requires comprehensive support. Social work case management programs must include well-trained medical professionals and support staff who have expertise with neonatal intensive care unit (NICU) patients and are familiar with the major challenges faced by this patient population.^7^ The combination of interdisciplinary support and individualized advocacy offered by a case management team promotes inclusive, socially-conscious care. A study team that leveraged qualitative and grounded theory methodology conducted focus groups and interviews with 18 families of low SES whose newborns received treatment in a California NICU. The results of the study highlighted several issues, such as a limited understanding of social work among patients with low SES and prejudice or reluctance of staff to address familial obstacles, the latter of which included involving Child Protective Services without conversing with families prior and not spending sufficient time to discuss relevant resources and needs.^8^ Social workers in NICUs provide support, increase knowledge of services, and advocate for the equitable treatment of patients.^9^ Case management has the potential to meet the social needs of patients and improve patient health literacy, including an understanding of costs and care plans.

Managed care organizations (MCOs) continue to play a vital role in mitigating healthcare costs. In a study of 365 parents of premature infants, only 26% of parents whose newborns were hospitalized in the NICU discussed costs of care with NICU staff and only 19% of parents discussed costs of care with their pediatrician.^10^ In a recent study, the CenteringPregnancy group demonstrated a novel approach to prenatal care involving group-based education. This method resulted in significant cost savings for CenteringPregnancy, with an estimated net savings of $67 293 reduction in NICU costs for 85 patients, or an average of approximately $792 per patient.^11^ To ensure equity in direct healthcare expenses, MCOs should ensure adequate dispersal and knowledge of resources for families of Medicaid-enrolled EP infants.

The current standard of care does not include many strategic clinical therapies and procedures capable of minimizing the occurrence of preterm comorbidities. Sammour and colleagues^12^ conducted a study that investigated the importance of early respiratory management, in which early continuous positive airway pressure and meticulous intubation standards were seen to prevent lung injury and bronchopulmonary dysplasia at first resuscitation. Additionally, surfactant therapy using less invasive administration techniques was associated with a lower risk of bronchopulmonary dysplasia when compared with the intubate-surfactant-extubate technique, the current standard of care.^13^ Bronchopulmonary dysplasia compromises respiratory function into adulthood and its risk can be lowered by use of inhaled corticosteroids, systemic hydrocortisone administration, and avoiding invasive ventilation when possible.^14^ Notably, ventilators and surfactant therapy can contribute to pneumonia. Oropharyngeal colostrum therapy, which is well-regarded for its immunoprotective and anti-inflammatory benefits, may significantly reduce the occurrence of ventilator-associated pneumonia.^15,16^ Coupled with case management and MCOs, the utilization of these therapies may collectively help reduce the burden of comorbidities and costs associated with high healthcare resource utilization.

Black newborns constitute a disproportionate share of preterm births and are born at a lower gestational age than White infants in aggregate. In a study conducted by Karvonen et al,^17^ Black infants experienced an elevated risk of developing bronchopulmonary dysplasia and other preterm comorbidities in addition to increased rates of hospital readmission. Racial and ethnic inequities related to premature birth and maternal-fetal outcomes are still prevalent despite advances in medical care, necessitating targeted interventions to reduce racial inequities in EP care and holistically across the national healthcare landscape. Revising and instituting socioeconomic policies that actionably advocate for vulnerable patient populations are an important step to addressing racial inequities in healthcare.^18^ Social and public policies that improve living conditions and environments to prevent illness and promote health have the potential for immense impact in reducing socioeconomic and racial/ethnic disparities in health.^19^ Evidence-based education, research, policy, and medical initiatives aimed at eliminating social and racial injustices must be appropriately evaluated at the local, state, and federal levels to reduce systematic marginalization and downstream consequences that affect the population health of those most vulnerable.

EP infant comorbidities, alongside the corresponding costs of relatively high use of healthcare resources, may be successfully reduced by strategies that are socioeconomically tailored. Skilled and empathetic patient navigators, social workers, case managers, and MCOs all have an important role to play in minimizing patient costs and ensuring the delivery of high-quality healthcare. We commend Mowitz and colleagues for their rigorous approach, nuanced analysis, and contribution to the field of health economics via their research of the premature infant population. Their findings are supported by robust statistical evidence and provide an excellent foundation for further policy action and future investigation into socioeconomic disparities among Medicaid-enrolled EP infants and vulnerable patient populations writ large.
